# The genomic stability regulator PTIP is required for proper chromosome segregation in mitosis

**DOI:** 10.1186/s13008-022-00081-4

**Published:** 2022-09-24

**Authors:** Fengxia Zhang, Mingxuan Wei, Haoran Chen, Liting Ji, Yan Nie, Jungseog Kang

**Affiliations:** 1 Arts and Science, New York University Shanghai, Shanghai, 200122 China; 2 Shanghai Institute for Advanced Immunochemical Studies, ShanghaiTech University, Shanghai, 201210 China; 3 NYU-ECNU Center for Computational Chemistry at NYU Shanghai, Shanghai, 200062 China

**Keywords:** Chromosome segregation, Mitosis, Genetic instability, Centrosome, DNA damage, PTIP

## Abstract

**Background:**

The Pax transcription activation domain-interacting protein (PTIP) is a nuclear protein that is an essential component of H3K4 methylation for gene activation in vascular, kidney, B cell, and adipocyte development. Furthermore, it plays a key role in genomic stability in higher eukaryotic cells. It binds to 53BP1 and antagonizes inappropriate homologous recombination for a proper DNA damage response. Interestingly, an early study reported mitotic defects after PTIP inactivation, but it is not clear whether PTIP directly facilitates mitotic processes.

**Results:**

Here, we showed that PTIP is essential for the mitotic integrity of HeLa cells. PTIP inactivation increases cell death during mitotic exit, which appears to result from direct mitotic defects. PTIP inactivation did not affect the G2M DNA damage checkpoint during interphase upon etoposide treatment. However, in mitosis, PTIP inactivation results in prolonged mitotic time, inefficient chromosome alignment, and increased cell death. Furthermore, PTIP localizes to the mitotic centrosome via BRCT domains at the C-terminus.

**Conclusion:**

This study reveals a novel function of PTIP in maintaining the genomic stability of higher eukaryotes during mitosis. Therefore, its deregulation, which occurs in various tumors, may destabilize the genome by introducing an abnormal DNA damage response, as well as erroneous chromosome segregation.

**Supplementary Information:**

The online version contains supplementary material available at 10.1186/s13008-022-00081-4.

## Backgound

Genetic stability is essential for the survival of multicellular organisms. The eukaryotic genome is accurately replicated in the S phase and divided into two daughter cells in the M phase. Any mistakes in these processes result in genetic instability, which leads to cell death or cancer [12, 17]. During mitosis, duplicated sister chromatids are separated into daughter cells by a dynamic mitotic spindle apparatus that begins to assemble at the centrosome during early mitosis [27]. Many centrosomic proteins facilitate proper spindle formation to ensure accurate chromosome alignment at the metaphase plate and sister chromatid separation during anaphase [1, 30]. Several mitotic kinases, including Aurora kinases and Polo kinases, are localized at the centrosome and coordinate the entire process to achieve accurate chromosome segregation [16, 25]. However, their intricate regulatory mechanism requires further clarification.

The Pax transcription activation domain-interacting protein (PTIP) is an important regulator of the genetic stability of higher eukaryotic cells [10, 24]. It was first identified via a two-hybrid screen for a Pax2 transcription activator [18]. It is essential for early mouse development [5], and its inactivation abolishes various cell differentiation programs during development [6, 8, 19, 22]. Because PTIP interacts with MLL methyltransferase, it is believed that PTIP activates Pax2-dependent gene expression by recruiting the MLL complex [7, 14, 26]. Interestingly, mass spectrometry analysis of PTIP immunoprecipitation revealed it also interacts with the PA1/53BP1-containing complex [7, 11]. The 53BP1 protein is known to function in the DNA double-strand break repair (DDR) pathway, such as the non-homologous DNA end-joining (NHEJ) repair at the G1 phase [33]. The 53BP1 mutant, which cannot bind to PTIP, displays severe defects in preventing erroneous homologous recombination repair [4, 23]. Therefore, PTIP plays an additional role in maintaining the genomic stability of higher eukaryotic cells through 53BP1, and its downregulation has been correlated with an aggressive tumor phenotype or poor prognosis [9, 13, 31]. It is important to note that PTIP has six BRCT domains, which are commonly observed in proteins involved in DNA damage repair and cell cycle regulation. Furthermore, an early study of PTIP showed that its inactivation greatly destabilizes the mitotic spindle apparatus, leading to cell death [5], but it is not clear whether these defects are due to accumulated DNA damages, decreased mitotic gene expression, or an absence of mitotic PTIP function.

Here, we hypothesized that PTIP directly regulated chromosome segregation during mitosis. We showed that PTIP is localized to the centrosome through the C-terminal BRCT domains and its inactivation delays metaphase chromosome alignment, which often leads to cell death. Therefore, PTIP may regulate centrosome dynamics to facilitate efficient chromosome segregation.

## Results

### PTIP is essential for mitotic progression and cell survival

**Fig. 1:**
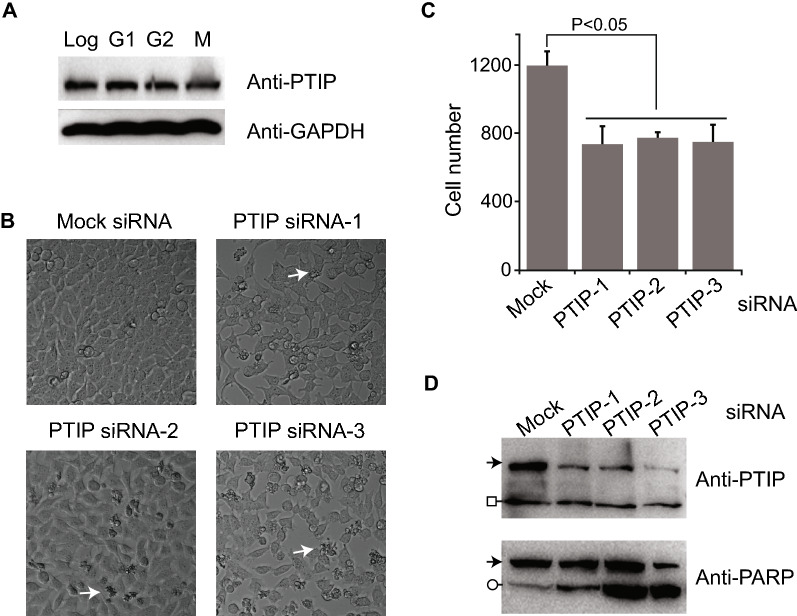
Proliferation of PTIP RNAi cells. **A** HeLa Tet-on cells were arrested by thymidine (G1), RO3306 (G2), or nocodoazole (M), and analyzed for PTIP protein level. **B** HeLa Tet-on cells were transfected with three different PTIP siRNA oligos for 36 h and DIC images were taken. White arrow indicates an example of blebbing cells. **C** Cells from **B** were counted by bright spot detection function of Nikon Eclipse software after staining DNA with Hoechst 33,342. Three replicates of treatments were used for analysis. P-value was calculated by student t-test, paired, two-tailed. **D** HeLa Tet-on cells were transfected with three different PTIP siRNA oligos for 36 h and total lysates of cells were resolved in SDS-PAGE and processed for western blot using PTIP antibody and PARP antibody. Arrows indicate full-length PTIP or PARP proteins. Circle indicates cleaved PARP and square indicates non-specific protein.

Previously, we carried out a two-hybrid screen of Mps1 mitotic kinase to understand how Mps1 regulates the chromosome segregation process, and we identified truncated PTIP (codon 423–1069) as a positive interactor of the Mps1 N-terminus (codon 1–517) (not published). We verified their interaction in vivo using a co-immunoprecipitation experiment (Additional file 1: Figure S1A). Mps1 binds to both PTIP-NM (codon 1–796) and PTIP-MC (codon 391–1069) but interaction to NM truncation is stronger. PTIP has been known to regulate gene expression and DNA damage repair during interphase. To investigate whether PTIP functions in mitosis, we first examined the level of PTIP protein at different cell cycle stages. PTIP protein abundance did not change during the different cell cycle stages, which is consistent with the result of an earlier study [5] (Additional file 1: Figure 1A, S1B). Next, we depleted PTIP in HeLa cells using three different siRNA oligos targeting different regions of PTIP and examined their phenotypes. As PTIP-knockout mice showed embryonic lethality [5], PTIP depletion by all three siRNA significantly decreased total cell numbers with more blebbing cells, which is a sign of apoptosis (Fig. 1B, C). Consistently, the level of cleaved PARP protein, a marker of apoptotic cell death, was increased in PTIP-depleted cells by all three siRNA, which indicated PTIP depletion allowed cells prone to apoptotic cell death (Fig. 1D). Since PTIP-3 siRNA oligos produced the most efficient knockdown of cells, we used PTIP-3 siRNA oligos for all the following depletion experiments.

**Fig. 2:**
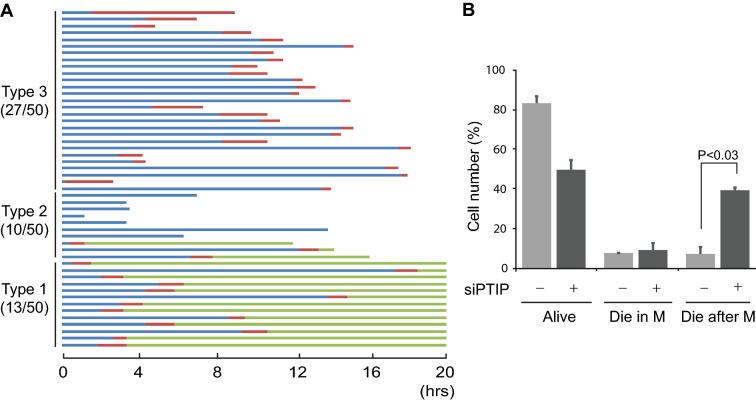
Cell fate after PTIP inactivation. **A** Hela Tet-on cells were treated with PTIP RNAi for 18 h and then subjected to live cell imaging for 20 h. 50 cells were counted for cell fate. Blue and green lines indicate interphase, and red lines indicate M phase. Type 1: cells survived after 20 h. Type 2: cells died in interphase. Type 3: cells died during mitosis. **B** HeLa Tet-on H2B-mCherry cells were transfected with PTIP siRNA oligo for 24 h and H2B-mCherrry images of live cells were taken for additional 20 h. Movies of about a hundred mitotic cells were analyzed for their fates in two independent experiments. P-value was calculated by student t-test, paired, two-tailed

To determine the exact cell cycle stage corresponding to cell death after PTIP depletion, we performed live-cell imaging. We first depleted PTIP using siRNA for 18 h and began live-cell imaging with phase-contrast microscopy for 20 h. The beginning of mitosis was determined by the onset of cell rounding, ending of mitosis by cytokinesis, and cell death by blebbing. Out of 50 tracked cells, 27 cells triggered apoptosis during mitosis (type 3), and 10 cells triggered apoptosis during interphase (type 2), suggesting that most PTIP-inactivated cells die in mitosis (Fig. 2A). Because it is not possible to distinguish specific mitotic phases from phase-contrast microscope images, we performed a similar experiment using the HeLa H2B-mCherry stable line and tracked cells using the H2B-mCherry signal to determine the specific stages of mitosis in which cells die. The beginning and ending of mitosis were recognized with the nuclear envelope breakdown and the anaphase, respectively. Almost 40% of all mitotic cells that we counted died either during cytokinesis or directly afterward (Fig. 2B). This result indicates that PTIP is essential for cell survival during late mitosis or early G1 stage.

### Mitotic defects in PTIP-depleted cells are not caused by DNA damage

**Fig. 3:**
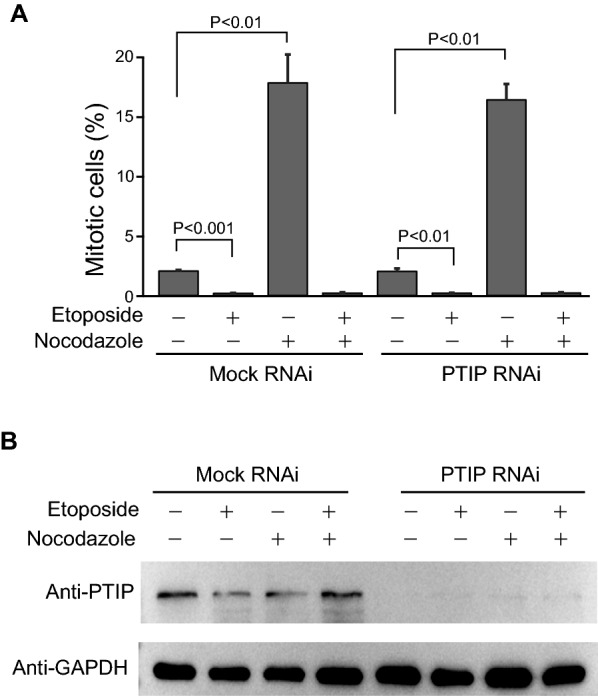
G2M DNA damage checkpoint of PTIP RNAi cells. **A** HeLa Tet-on cells were treated with mock or PTIP RNAi for 24 h and DNA damages were induced by etoposide for 24 h. Then, cells were treated with nocodazole for 5 h and fixed for IF. Mitotic cells were counted from DNA images containing about 1000 cells. Statistics of three independent experiments was shown. P-value was calculated by student T test, paired, two tailed. **B** HeLa Tet-on cells were treated with mock or PTIP RNAi for 24 h and DNA damages were induced by etoposide for 24 h. Then, cells were treated with nocodazole for 5 h. Total lysates were resolved by SDS-PAGE and processed for western blot

PTIP inactivation has demonstrated its hypersensitivity to IR-induced DNA double-strand breaks [5, 11, 15]. We first investigated whether spontaneous DNA damage during interphase by PTIP inactivation might indirectly interfere with the integrity of mitotic processes. Thus, we examined the integrity of the G2M DNA damage checkpoint after PTIP depletion. If PTIP depletion does not affect the G2M DNA damage checkpoint, cells with DNA damage are less likely to undergo mitosis. For the G2M checkpoint assay, we treated the cells with two drugs, etoposide and nocodazole, and examined the mitotic index. If the G2M checkpoint is functional, mitotic cells do not accumulate after consecutive drug treatment. As expected, consecutive drug treatment did not increase mitotic index in mock-depleted cells, as well as PTIP-depleted cells, suggesting that PTIP depletion does not affect G2M DNA damage checkpoint signaling (Fig. 3A, B). Therefore, mitotic defects in PTIP-depleted cells are unlikely caused by DNA damage occurring during the interphase. However, we cannot rule out the possibility that a small fraction of cells may experience minor DNA damage after PTIP RNAi and pass through mitosis.

### PTIP inactivation interferes with chromosome alignment during mitosis

**Fig. 4:**
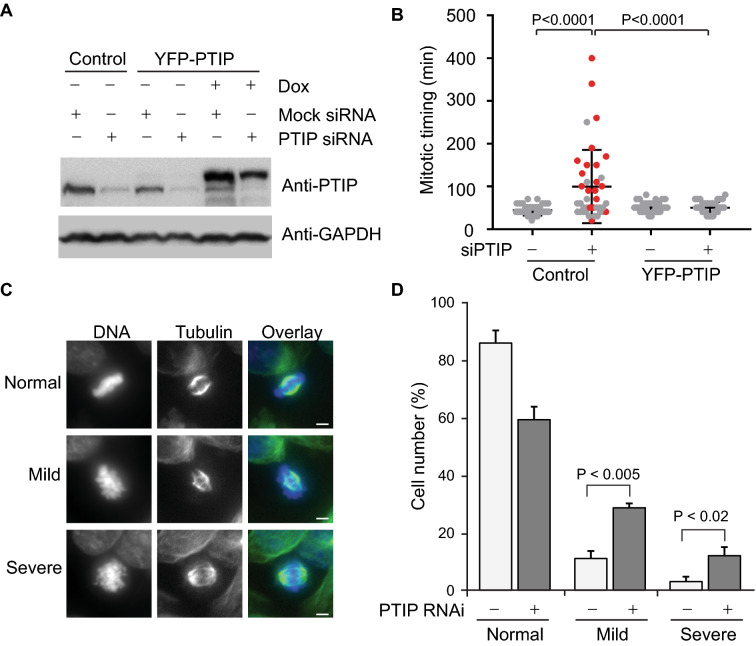
YFP-PTIP stable line for mitotic process. **A** RNAi resistant YFP-PTIP was induced by doxycycline from HeLa Tet-on stable line and PTIP siRNA oligos were transfected for 24 h. Total lysates of stable line were resolved in SDS-PAGE and processed for western blot using PTIP antibody and GAPDH antibody. **B** HeLa Tet-on control or YFP-PTIP stable cell line was treated with PTIP RNAi for 18 h and subjected to live-cell imaging for 20 h. About 50 mitotic cells were examined for mitotic time. Red dots indicate cells that died in or just after mitosis. P-value was calculated by student t-test, paired, two-tailed. **C** HeLa Tet-on cells were first arrested at G1 by thymidine and transfected with PTIP siRNA oligos. Then, they are released from G1 block for 10 h and arrested at metaphase by MG132 for 1 h. Representative images of normal alignment or mild/severe misalignment were shown. Normal cells mean metaphase cells with all aligned chromosomes at the metaphase plate. Mild cells mean metaphase cells with the majority of chromosome aligned at the metaphase plate but the minority of chromosomes proximally located away from the metaphase plate. Severe cells are similar to the mild cells except misaligned chromosomes farther away from the metaphase plate. Size bar, 5 µm. **D** Statistics of panel **C** was shown. About 100 mitotic cells were counted in three replicates. P-value was calculated by student t-test, paired two-tailed

Because siRNA inactivation often exhibits an off-target effect, we wanted to confirm that the cell death phenotype did not result from an off-target effect. Thus, we generated HeLa cells expressing RNAi-resistant YFP-PTIP using a tetracycline-regulated promoter and examined PTIP abundance after siRNA transfection. Endogenous PTIP was efficiently depleted by siRNA transfection. Exogenous YFP-PTIP was also depleted by siRNA to some degree, but the remaining amount was comparable to the endogenous PTIP levels of control HeLa cells (Fig. 4A). Next, we examined the mitotic time of the established cell lines and their viability after PTIP depletion (Fig. 4B). Many control cells prolonged mitosis and eventually died by PTIP depletion, but YFP-PTIP stable cells grew normally and finished mitosis in approximately 50 min, suggesting that the cell death phenotype caused by PTIP depletion is an on-target effect.

Next, we investigated whether PTIP regulates the chromosome segregation process, since its depletion prolongs mitotic time. Thus, we arrested cells at metaphase with MG132 for a short time and examined how efficiently chromosomes aligned at the metaphase plate. Mock RNAi cells mostly aligned chromosomes in the metaphase plate after 1 h of MG132 arrest, but PTIP RNAi cells could not align chromosomes efficiently, frequently exhibiting lagging chromosomes (Fig. 4C, D). These data demonstrate that PTIP facilitates efficient chromosome alignment in metaphase cells.

### A fraction of PTIP is localized to the centrosome

**Fig. 5:**
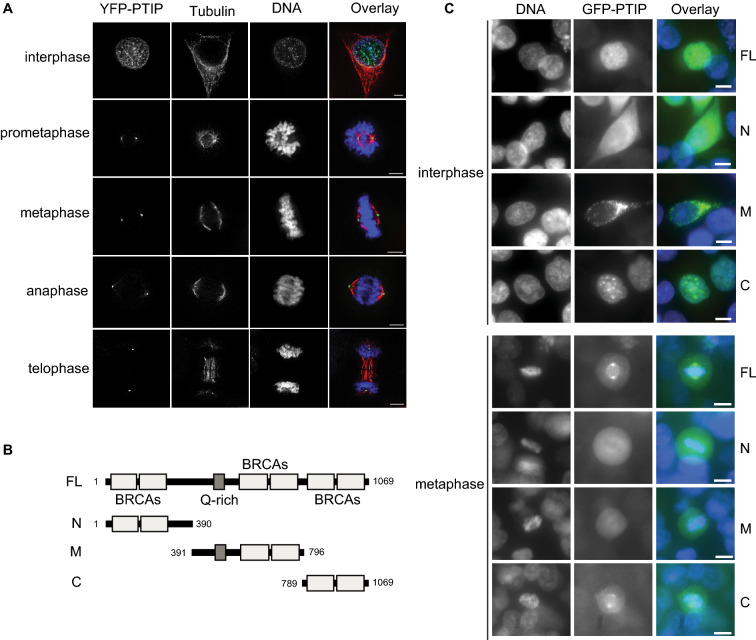
Localization of PTIP near centrosomes. **A** Cell cycle-specific localization. YFP-PTIP was induced by doxycycline from HeLa Tet-on stable cell line and its localization was examined by immunofluorescence using GFP antibody. Microtubules were shown by β-tubulin antibody and DNA by Hoechst 33342. Size bar, 5 µm. **B** Domain structures of PTIP full-length or truncations were illustrated. **C** Domain-specific localization. GFP-PTIP truncations are expressed in HeLa Tet-on cells and their localization in M-phase or interphase cells were examined by immunofluorescence using GFP antibody. DNA was shown by Hoechst 33342 staining. Size bar, 10 µm

PTIP has been shown to be localized to the nucleus during interphase [18], but its mitotic localization has not been thoroughly studied. Thus, to determine whether PTIP is localized to specific mitotic structures during mitosis, HeLa cells expressing YFP-PTIP were processed for indirect immunofluorescence using GFP antibodies. Interestingly, we observed abundant mitotic cytoplasmic localization and weak mitotic spindle localization (data not shown). To verify its weak spindle localization, we removed most of the cytoplasmic fraction through pre-extraction before cell fixation and performed immunofluorescence analysis (Fig. 5A). YFP-PTIP was clearly localized to the centrosome during the entire M phase in the established cells, but not in the control cells. To determine which region of PTIP is sufficient for the centrosome localization, we constructed three GFP-PTIP-N, -M, or -C truncations, together with GFP-PTIP-FL, and examined their cellular localization using the pre-extraction method (Fig. 5B, C). Interestingly, PTIP-FL and -C truncation exhibited a similar localization pattern, specific nuclear foci during interphase, and specific centrosomes during mitosis, indicating that the two C-terminal BRCT domains play an important role in their cellular localization. PTIP-N or -M truncations did not show specific localization, except for some cytoplasmic foci of M truncation in the interphase. These data strongly suggest that PTIP facilitates chromosome segregation at centrosomes.

## Discussion

Here, we have shown that the genetic stability regulator PTIP is localized to the centrosome of mitotic cells and facilitates chromosome segregation. Previously, PTIP was shown to function in both Pax2-dependent transcriptional regulation and NHEJ DDR during interphase [10, 24], but it is not clear whether PTIP also functions in mitotic processes. Since we identified PTIP as a binding partner of Mps1, an important mitotic kinase, we investigated its putative roles in mitosis. Our study shows that the prominent phenotype of PTIP inactivation is mitotic cell death, which results from abnormal chromosome segregation. Cells experiencing long mitosis tend to die at the G1 phase due to increased p53 levels [28, 29]. However, cell death via PTIP inactivation is less likely to be caused by increased p53 levels, since HeLa cells do not have functional p53 gene products. Thus, it will be important to study what kind of mitotic damage accumulates after PTIP inactivation and what factors mediate the signaling pathway, resulting in apoptotic cell death during mitosis.

PTIP is localized to specific nuclear foci during interphase, but it is evenly distributed throughout the cytoplasm during mitosis. Interestingly, we observed additional subcellular localization to the centrosome and mitotic spindles near the centrosome after removing an abundant cytosolic fraction of PTIP using the pre-extraction method. Centrosome localization has been observed with GFP antibodies for YFP-PTIP and Myc antibodies for Myc-PTIP, suggesting that it is not an artifact of non-specific antibodies. However, we could not detect PTIP in the centrosomes using commercial or homemade PTIP antibodies. This might result from masking of the antibody-binding domain by other binding proteins or from its low abundance at the centrosome, which is supported by the fact that overexpression of GFP fusion protein overcomes these detection limitations. Since a small fraction of PTIP at the centrosome is sufficient for mitotic function, PTIP may play a catalytic role in regulating chromosome segregation. In this regard, it is interesting to note that PTIP interacts with the MLL3/4 complex to regulate histone methylation [7, 26]. Therefore, it is intriguing that PTIP may bring the MLL3/MLL4 complex to centrosomic proteins for methylation. Furthermore, MLL and its binding partners are localized to the mitotic spindle apparatus [2, 21, 32], and RNAi inactivation of members of the methyltransferase family, SETA or SETB, generates similar mitotic defects [3].

Our finding that PTIP is localized to the centrosome is consistent with the centrosomic localization of Mps1, its binding partner [20]. *MPS1* was first identified using a yeast genetic screen as an essential regulator of both spindle pole duplication and the spindle assembly checkpoint, and it is highly conserved from yeast to humans. Since Mps1 interacts with PTIP, and both proteins localize to the centrosome, it would be interesting to study whether Mps1 phosphorylates PTIP to regulate its function at the centrosome, as well as other mitotic structures, to maintain the genetic stability of higher eukaryotic cells.

## Conclusion

Since its identification as a Pax2 transcription-interacting protein, multiple roles of PTIP in the proliferation and genomic stability of higher eukaryotic cells have been reported. Many questions still need to be answered to understand how PTIP functions in determining cell differentiation programs and maintaining genomic stability. Identifying its binding partners in different cell cycle stages and contexts may provide a better understanding of its delicate functional regulation. Since the genetic stability of higher eukaryotic cells depends on accurate genome replication and segregation, investigation of dual regulators, such as PTIP, will further our understanding of how cancers utilize genomic instability for their benefits.

## Methods

### Cell culture and transfection

HeLa Tet-on cells (Clontech) were grown at 37 °C in DMEM (Hyclone) supplemented with 10% fetal bovine serum (Sorfa life science) in a humid atmosphere with 5% CO_2_. For mitotic arrest, cells were treated with 330 nM nocodazole (Sigma) for 12–14 h. For chromosome alignment assay, 10 μM MG132 (Sigma) was treated for 1–2 h. For DNA damage experiment, 10 μM etoposide (Sigma) was treated for 10 or 24 h. For the generation of stable cell lines, HeLa Tet-on cells were transfected with pTRE2-hyg vectors encoding siRNA-resistant wild-type YFP-PTIP transgenes. Clones of cells were selected in regular media containing 200–400 μg/ml of hygromycin (Mesgen) and maintained in media with 100 μg/ml of hygromycin. 5 μg/ml of doxycycline (Mesgen) was used for induction of PTIP expression. For RNAi experiment, cells were transfected with siRNA oligonucleotides (Genepharma) using Lipofectamine RNAiMAX (Invitrogen) for 24–48 h. The sequences of the PTIP-1, − 2, − 3 siRNAs were 5′- acacugaggaauauuacuaaa-3′, 5′-ccagcagggacauacaaauaa-3′, 5′- gcccaagucuuuccacuauaa-3′, respectively. The mutagenized PTIP sequence to make it resistant to PTIP-3 siRNA is 5′-gcccttccctgagtactat-3′. Plasmid transfection was carried out by Effectene reagent (Qiagen) according to the manufacturer’s instructions.

### Antibodies and coimmunoprecipitation

The following primary antibodies were used for immunofluorescence or western blot: anti-β-tubulin (CWBIO, 1:500), anti-GFP (Proteintech, 1:300), anti-Myc (Proteintech, 1:1000), anti-HA (Roche, 1:1000), anti-GAPDH (Mesgen, 1:5000), and anti-Actin (Mesgen, 1:1000), and anti-PTIP (this study, 1:2000). Anti-PTIP antibody was generated by immunizing rabbits with purified His_6_-PTIP ∆^2^ truncation (codon 202–370). The following secondary antibodies were used for immunofluorescence or western blot: goat anti-mouse IgG, HRP conjugate (Proteintech, 1:5000), goat anti-rabbit IgG, HRP conjugate (Proteintech, 1:5000), goat-anti-rabbit IgG, FITC conjugated (CWBIO, 1:300), goat-anti-rabbit IgG, TRIRC conjugated (CWBIO, 1:300), goat-anti-mouse IgG, FITC conjugated (CWBIO, 1:300), and goat-anti-mouse IgG, TRITC conjugated (CWBIO, 1:300).

For coimmunoprecipitation, mitotic cells coexpressing HA-Mps1 and Myc-PTIP were incubated with lysis buffer (25 mM Tris–HCl, pH 7.4, 150 mM NaCl, 1% NP-40, 1 mM EDTA, 5% glycerol) supplemented with protease inhibitor cocktail (Sigma) and phosphatase inhibitor cocktail (Mesgen) on ice for 5 min. Cell lysates were retrieved by scraping and cleared by centrifugation. The supernatants were incubated with anti–c-Myc magnetic beads (Pierce) at 4 °C for 2 h and proteins bound to beads were released with SDS loading buffer, resolved in SDS–PAGE, and analyzed by Western blotting. For examining protein abundance, cells were collected by trypsin and lysed by sonication in SDS-PAGE loading buffer. Total lysates were resolved in SDS-PAGE and analyzed by western blotting.

### Immunofluorescence imaging and counting

Cells grown on 96-well imaging plate (Corning) or cover glass in 12-well plate were pre-extracted in PHEM buffer (60 mM PIPES, 25 mM HEPES, 10 mM EGTA, 4 mM MgSO_4_, pH 7.0) containing 0.05% digitonin for 5 min and fixed by 4% paraformaldehyde in PHEM buffer at room temperature for 10 min. Cells were then permeabilized with PBST (phosphate buffered saline containing 0.1% Triton X-100) and blocked with PBST containing 3% BSA for 30 min. Primary antibodies were incubated at room temperature for 2 h and secondary antibodies for 1 h. DNA was stained by Hoechst 33,342 (1 µg/ml) for 5 min. Cell images were acquired by a 20X objective lens in a Nikon Eclipse Ti-E microscope and processed with ImageJ (NIH). For time-lapse imaging, HeLa Tet-on YFP-PTIP cells or H2B-mCherry cells were recorded every 10 min for a total duration of 24 h with a 10X objective in a Nikon Eclipse Ti-E microscope equipped with a temperature- and CO_2_-controlled stage incubation unit (Okolab).

For cell counting, cells were first grown onto 96-well imaging plates. Cells were then fixed with 70% ethanol for 2 h at room temperature and stained with PBS containing 0.1% Triton X-100 and Hoechst 33342 (1 µg/ml) for 5 min. Three cell images per condition were acquired by a 4X objective lens in a Nikon Eclipse Ti-E microscope and total cells in the image were counted by bright spot detection function in Nikon Eclipse software. Three biological replicates were used for statistics.

## Supplementary Information

**Additional file 1: Figure S1.** **A** Protein interaction between Mps1 and PTIP. HeLa Tet-on cells were cotransfected with HA-Mps1 and Myc-PTIP truncations for 24 hrs and arrested at the M-phase with nocodazole for 15 hrs. Myc-PTIP truncations were pulled down by Myc beads. Total lysates and IPed fractions were resolved by SDS-PAGE and processed for western blot. **B** Cell cycle synchronization. HeLa Tet-on cells were arrested by thymidine (G1), RO3306 (G2), or nocodoazole (M). Their images were taken by a phase-contrast microscope (upper) and their DNA contents were analyzed by FACS (lower).

## Data Availability

The data that support the findings of this study are available from the corresponding author upon reasonable request.

## Abbreviations

PTIP: Pax Transcription activation domain interacting protein
53BP1: Tumor suppressor p53-binding protein 1
BRCA1: BReast cancer type 1
BRCT: BRCA1 C-terminus
MLL: Mixed lineage leukemia
PA1: PTIP associated protein 1
DDR: DNA double-strand break repair
NHEJ: Non-homologous DNA end joining
HR: Homologous recombination
GFP/YFP: Green fluorescent protein/yellow fluorescent protein
RNAi: RNA interference
